# Overexpression of the *OsIMP* Gene Increases the Accumulation of Inositol and Confers Enhanced Cold Tolerance in Tobacco through Modulation of the Antioxidant Enzymes’ Activities

**DOI:** 10.3390/genes8070179

**Published:** 2017-07-20

**Authors:** Rong-Xiang Zhang, Li-Jun Qin, De-Gang Zhao

**Affiliations:** 1 Key Laboratory of Plant Resources Conservation and Germplasm Innovation in Mountainous Region, Ministry of Education, Institute of Agro-Bioengineering and College of Life Sciences, Guizhou University, Guiyang 550025, China; leequine_chin@126.com; 2 State Key Laboratory Breeding Base of Green Pesticide and Agricultural Bioengineering, Guizhou University, Guiyang 550025, China; 3 Guizhou Academy of Agricultural Sciences, Guiyang 550025, China; 4 College of Chemistry and Life Science, Guizhou Education University, Guiyang 550018, China; rxzhang@gznc.edu.cn

**Keywords:** rice, l-*myo*-inositol monophosphatase, l-*myo*-inositol, cold tolerance, antioxidant enzymes

## Abstract

Inositol is a cyclic polyol that is involved in various physiological processes, including signal transduction and stress adaptation in plants. l-*myo*-inositol monophosphatase (IMPase) is one of the metal-dependent phosphatase family members and catalyzes the last reaction step of biosynthesis of inositol. Although increased IMPase activity induced by abiotic stress has been reported in chickpea plants, the role and regulation of the *IMP* gene in rice (*Oryza sativa* L.) remains poorly understood. In the present work, we obtained a full-length cDNA sequence coding IMPase in the cold tolerant rice landraces in Gaogonggui, which is named as *OsIMP*. Multiple alignment results have displayed that this sequence has characteristic signature motifs and conserved enzyme active sites of the phosphatase super family. Phylogenetic analysis showed that IMPase is most closely related to that of the wild rice *Oryza brachyantha*, while transcript analysis revealed that the expression of the *OsIMP* is significantly induced by cold stress and exogenous abscisic acid (ABA) treatment. Meanwhile, we cloned the 5’ flanking promoter sequence of the *OsIMP* gene and identified several important *cis*-acting elements, such as LTR (low-temperature responsiveness), TCA-element (salicylic acid responsiveness), ABRE-element (abscisic acid responsiveness), GARE-motif (gibberellin responsive), MBS (MYB Binding Site) and other *cis*-acting elements related to defense and stress responsiveness. To further investigate the potential function of the *OsIMP* gene, we generated transgenic tobacco plants overexpressing the *OsIMP* gene and the cold tolerance test indicated that these transgenic tobacco plants exhibit improved cold tolerance. Furthermore, transgenic tobacco plants have a lower level of hydrogen peroxide (H_2_O_2_) and malondialdehyde (MDA), and a higher content of total chlorophyll as well as increased antioxidant enzyme activities of superoxide dismutase (SOD), catalase (CAT) and peroxidase (POD), when compared to wild type (WT) tobacco plants under normal and cold stress conditions.

## 1. Introduction

Rice is one of the most important cereal crops in developing countries, and is the main source of food for nearly half of the population in the world [1]. Recently, with global environmental deterioration and abnormal climate conditions, various abiotic stresses have severely affected the production of rice. In particular, rice production is affected by low temperature, which is considered as the major limiting factor that affects the production and geographical distribution of rice because of its primordial origin from tropical and subtropical areas, leading to its extreme sensitivity to chilling. Therefore, the development of cold-tolerant rice cultivation is an important goal of rice breeding programs. Over the past several decades, researchers have made great efforts to enhance the resistance to cold stress in rice by conventional breeding strategies, but they have seemed to not be effective because of the complex traits of the cold stress response in plants [2]. 

With the development of the modern techniques of molecular biology, many novel genes improving the cold tolerance have been cloned and characterized in rice. Meanwhile, the rapid advancement in genetic engineering technology, together with precise and efficient DNA transformation methods, have made it possible to acquire genetically modified rice plants with improved cold tolerance [3,4,5,6]. 

*Myo*-inositol (MI), a cyclic polyol, is found ubiquitously in the biological kingdom, whose de novo biosynthesis undergoes three main reactions from the precursor d-glucose. Initially, d-glucose is phosphorylated to form d-glucose-6-P by the enzyme hexokinase, then l-*myo*-inositol 1-phosphate synthase (MIPS) catalyzes the conversion of the d-glucose-6-P to l-*myo*-inositol 1-phosphate. Finally, l-*myo*-inositol 1-phosphate is dephosphorylated to produce *myo*-inositol by l-*myo*-inositol monophosphatase (IMPase) [7]. In higher plants, *myo*-inositol plays diverse roles in growth development, maintaining the membrane components, being osmoprotectants and part of the environmental stress response [8,9]. For example, *myo*-inositol participates in programmed cell death and pathogen resistance mediated by salicylic acid in *Arabidopsis* [10,11]. Furthermore, a significant increase in MI content has been reported in plants under cold or salt stress conditions, suggesting that MI may be involved in protecting plants from abiotic stress injuries [12,13]. Furthermore, many crucial metabolites derived from MI, e.g., ononitol, pinitol, phytate, phosphatidylinositol, galactinol and the raffinose family of oligosaccharides (RFOs), have been confirmed to participate in phosphate storage, auxin physiology regulation, cellular signaling and stress adaptation in plants [14,15,16,17,18,19]. 

Unlike the enzyme MIPS that has been broadly studied, another key enzyme IMPase involved in MI biosynthesis has rarely been considered in research. The enzyme IMPase catalyzes the dephosphorylation of *myo*-inositol 1-phosphate during MI biosynthesis. Additionally, IMPase also dephosphorylates other inositol phosphate compounds to regenerate inositol [20]. Therefore, IMPase is essential for both biosynthesis and recycling of inositol as well as being a critical regulatory node in the inositol metabolism network.

The IMPase protein is a member of the phosphatase superfamily with lithium-sensitivity and wide substrate specificity [21]. In higher plants, the IMPase coding genes were cloned and characterized in *Arabidopsis* [22], barley [23] and tomato [24]. Biochemical studies have also revealed that IMPase also catalyzes the dephosphorylation of l-galactose 1-phosphate and was a bi-functional enzyme as it participates in the biosynthesis of ascorbic acid [25]. Although the expression pattern and role of the *IMP* gene in the developmental or stress-response processes in many plant species have been generally discussed, its functions are seldom studied [26,27]. We previously had analyzed the cold-induced genes from cold-tolerant rice landrace varieties in Gaogonggui in response to cold stress and identified a differentially expressed *IMP* gene through the subtractive complementary DNA (cDNA) hybridization technique. However, the regulatory role of the *IMP* gene in the rice varieties is obscure. Given that the transcript level of the *IMP* gene was strongly induced by cold stress in Gaogonggui, we initially proposed that *IMP* may play a critical role in low temperature stress responses. Therefore, the aims of this study were: (1) to further investigate the response of the *OsIMP* gene to cold stress and exogenous abscisic acid (ABA) treatment; (2) to study the cold tolerance of transgenic tobacco plants overexpressing the *OsIMP* gene; (3) to test how the *OsIMP* gene can regulate cold tolerance in rice by analyzing the biochemical indicators in transgenic tobacco plants. Here, we isolated a full-length cDNA sequence of the *IMP* gene, named as *OsIMP*. The expression profile analysis suggested that the transcript of this gene was not only induced by the cold stress but also by exogenous ABA. Furthermore, transgenic tobacco plants overexpressing *OsIMP* exhibited significantly improved cold tolerance in comparison to wild type plants. Our results indicated that the *OsIMP* gene had a potential in genetically improving tolerance to cold stress in rice.

## 2. Results

### 2.1. Cloning and Bioinformatic Analysis of the OsIMP Gene

**Figure 1:**
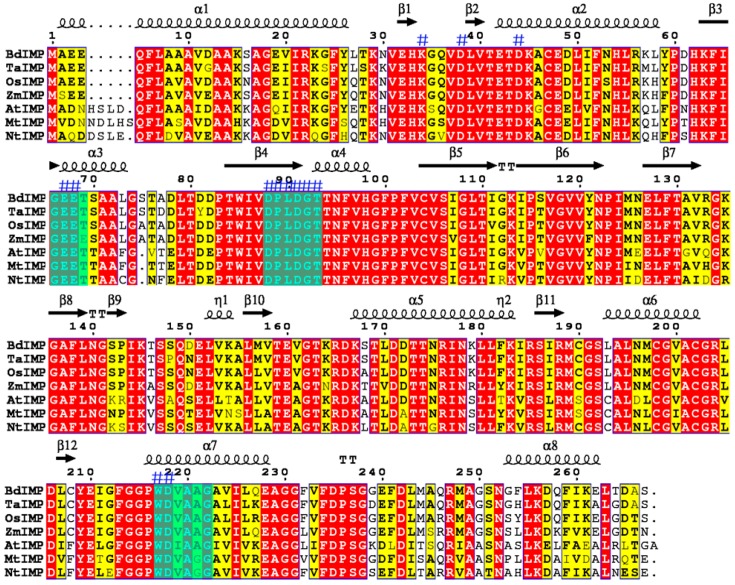
Multiple sequence alignments of l-*myo*-inositol monophosphatase (IMPase) from other plant species. Flat figure shows the sequences of IMPase from several plants. The second structure element symbols presented on the top (squiggles are α helices, arrows β strands and TT letters beta turns), identical and similar residues are boxed in red and yellow, respectively. Three characteristic signature motifs (GEET, DPLDGT and WDXAAG) of the phosphatase super family are boxed by purple colours. The enzyme active sites (i.e., lysine[K]^34^, aspartate[D]^38, 44, 88, 91, 218^, glutamic acid[E]^67, 68^, proline[P]^89^, leucine[L]^90^, glycine[G]^92^, threonine [T]^93^ and tryptophan[W]^217^) are marked by blue “#”. BdIMP, *Brachypodium distachyon* IMP; TaIMP, *Triticum aestivum* IMP; OsIMP, *Oryza sativa* (Gaogonggui) IMP; ZmIMP, *Zea mays*; AtIMP, *Arabidopsis thaliana* IMP; MtIMP, *Medicago truncatula* IMP; NtIMP, *Nicotiana tomentosiformis* IMP.

**Figure 2:**
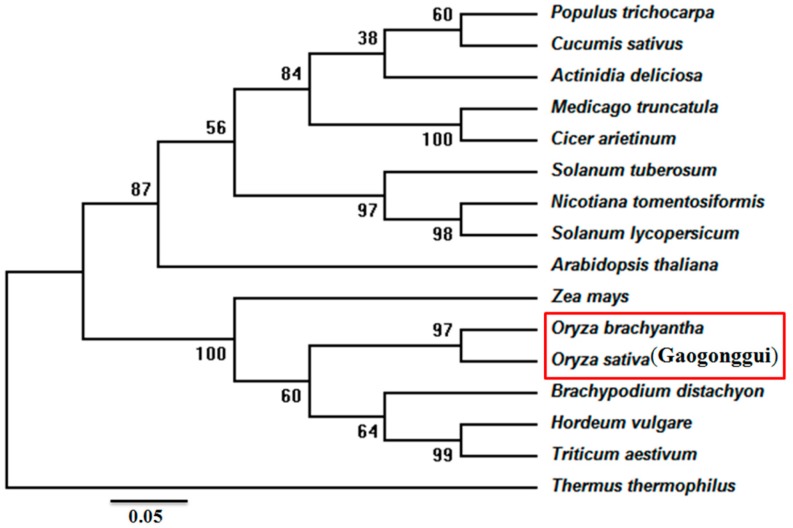
Phylogenetic analysis of the IMPase in rice cultivation in Gaogonggui and other plant species. The statistical reliability of individual nodes of the tree is assessed by bootstrap analyses with 1000 replications, and the bar represents the branch length equivalent to 0.05 amino acid changes per residue.

An 898 bp cDNA fragment was obtained from rice seedlings in Gaogonggui by reverse transcription–polymerase chain reaction (RT-PCR) (Figure S1a), subsequently validated by DNA sequencing. The putative gene was named as *OsIMP*, which includes an 801 bp open reading frame (nucleotide position 20–820) encoding a polypeptide of 267 amino acids with a predicted molecular mass of 29 kDa and an isoelectric point of 5.44. The multiple alignment results displayed that the deduced amino acid sequence of OsIMP shares a high sequence similarity with other sequences encoding IMPase from *Oryza brachyantha* (XP_006650302.1), *Nicotiana tomentosiformis* (XP_009618 414.1), A*rabidopsis thaliana* (AAM62772.1), *Medicago truncatula* (XP_003594 243.1), *Zea mays* (NP_001149505.1) and *Triticum aestivum* (AAS19203.1). There are three characteristic signature motifs (DPLDGT, WDXAAG and GEET) and a search of the CDD (conserved domain database) [28] showed that these proteins contain common conserved domains and enzyme active sites as marked by “#” (Figure 1). To compare the molecular evolutionary relationships of IMPase with that of other species, we downloaded 15 sequences encoding IMPase from plants and bacteria by BLAST searches from the National Center for Biotechnology Information (NCBI) database, and the phylogenetic analysis indicated that the IMPase in Gaogonggui is most closely related to that of the wild rice *Oryza brachyantha* (Figure 2). 

### 2.2. Transcription of the OsIMP Gene Was Induced by the Cold Stress and ABA Treatment

**Figure 3:**
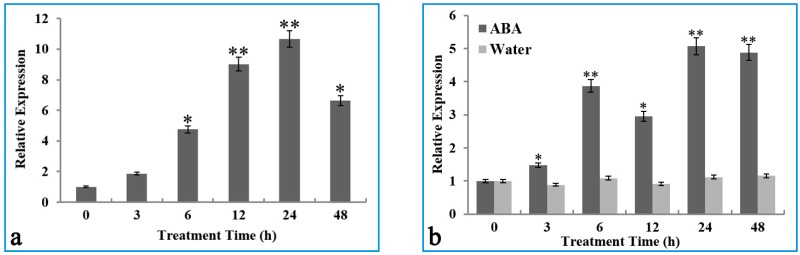
Quantitative reverse transcription (qRT)-PCR analysis of *OsIMP* gene transcript levels: (**a**) under cold stress (4 °C) and (**b**) in the presence of exogenous abscisic acid (ABA). The *actin* and *UBQ5* genes were used as an internal reference. Data are the mean ± SE of three biological replicates, while asterisks indicate significant differences (* *p* < 0.05, ** *p* < 0.01) in comparison with control.

To evaluate the expression patterns of the *OsIMP* gene responding to cold stress and ABA treatment, the transcript levels of the *OsIMP* gene in Gaogonggui seedlings were detected by quantitabive Real Time PCR (qRT-PCR). For cold stress treatment, a dramatic increase in the transcript level of the *OsIMP* gene was observed after 6 h of cold treatment, which climbed to the peak at the 24 h time point (Figure 3a). Meanwhile, it was found that ABA treatment induced a significant increase in transcript level of the *OsIMP* gene after 3 h of treatment, which gradually elevated until 24 h (Figure 3b). The results strongly support that cold stress and ABA treatment trigger the transcript of the *OsIMP* gene in rice variety Gaogonggui, with the *OsIMP* gene possibly possessing potential function for cold resistance. 

### 2.3. Amplification of OsIMP Gene Promoter and Cis-Acting Elements Prediction

To explore the underlying molecular mechanisms by which cold stress and ABA treatment regulate the transcription of the *OsIMP* gene, we obtained the 2200 bp promoter sequence upstream to the start codon of the *OsIMP* gene. Using PlantCARE and PLACE online tools, the promoter fragment was analyzed for *cis*-acting regulatory elements and binding sites of various transcriptional factors. Furthermore, a large number of critical TATA and CAAT box sequence elements, several important *cis*-acting regulatory elements involved in phytohormones and abiotic stresses were also found (Table 1). These include the Skn-1-motif, TC-rich repeats, TGA-element, TCA-element, ABRE-element, GARE-motif, LTR (low-temperature responsiveness), MBS (MYB Binding Site) and HSE (*cis*-acting element involved in heat stress responsiveness).

### 2.4. Plasmid Construction, Generation and Confirmation of the OsIMP Transgenic Tobacco Plants 

Restriction analysis and DNA sequencing results confirmed that we successfully constructed a plant binary vector overexpressing *OsIMP* gene (Figure S1b,c). The *Agrobacterium* strain LBA4404 harboring the pTCK303–OsIMP construct was used for the transformation of *Nicotiana tabacum* var. K326, while the hygromycin-resistant tobacco seedlings were verified by PCR amplification of the hygromycin gene. Out of 15 plants initially selected, 13 plants showed PCR amplification of 610 bp of the hygromycin gene (Figure S1d).

### 2.5. Overexpression of the OsIMP Gene Increased Inositol Content in Tobacco Plants

**Figure 4:**
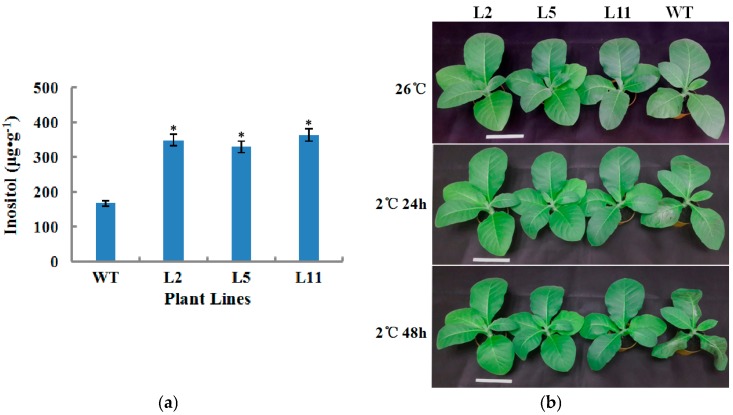
*OsIMP* transgenic lines exhibit improved cold tolerance. (**a**) Measurement of inositol in the leaf of wild type (WT) and *OsIMP* over-expression lines (L2, L5 and L11). Data are the mean ± SE of three biological replicates; asterisks indicate significant differences (* *p* < 0.05, ** *p* < 0.01) in comparison with wild type. (**b**) Phenotypes of seedlings of WT and transgenic tobacco plants (L2, L5 and L11) exposed to cold stress condition (2 °C) for 48 h. The scale bar represents 10 cm.

In an attempt to assess the effect of overexpressing the *OsIMP* gene on the biosynthesis of inositol in transgenic tobacco plants, T_1_ generation transgenic tobacco plant lines L2, L5 and L11 were chosen for analyzing the content of inositol. Results indicated that transgenic tobacco plants accumulate significantly more inositol than WT (wild type) tobacco plants (Figure 4a) and were selected for further functional validation for cold stress resistance.

### 2.6. Transgenic Tobacco Plants Exhibited Improved Tolerance to Cold Stress

**Figure 5:**
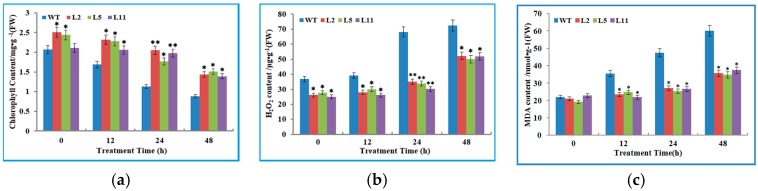
Accumulation of (**a**) chlorophyll, (**b**) H_2_O_2_ and (**c**) malondialdehyde (MDA) in the WT and transgenic lines (L2, L5 and L11) under cold stress conditions (2 °C) at different time points. Data are the mean ± SE of three biological replicates, while asterisks indicate significant differences (* *p* < 0.05, ** *p* < 0.01) in comparison with the wild type.

**Figure 6:**
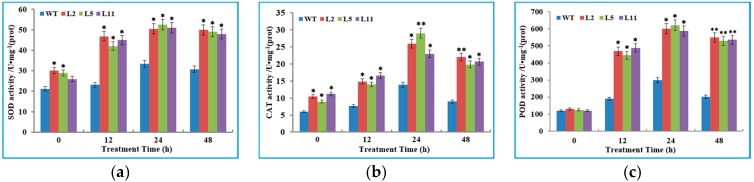
Analysis of antioxidative enzyme activity in the WT and transgenic lines (L2, L5 and L11) undergoing cold stress (2 °C) at different time points: (**a**) superoxide dismutase (SOD), (**b**) catalase (CAT) and (**c**) peroxidase (POD). Data are the mean ± SE of three biological replicates; asterisks indicate significant difference (* *p* < 0.05, ** *p* < 0.01) in comparison with wild type.

Three transgenic lines L2, L5 and L11, together with the wild type tobacco plants, were exposed to the low temperature of 2 °C for 48 h. The results showed that the wild type plants were slightly injured after 24 h of cold treatment, seriously injured when treated for 48 h. This was shown as the leaves displayed extensive wilting, although the transgenic lines were hardly injured (Figure 4b) and still healthy. Simultaneously, we analyzed the contents of total chlorophyll, H_2_O_2_ and malondialdehyde (MDA) in WT and transgenic tobacco plants at normal (26 °C) and cold temperature conditions. It was observed that the cold stress perturbed the synthesis of chlorophyll in both WT and transgenic tobacco plants. However, transgenic T_1_ plant lines showed a significantly higher content of chlorophyll than WT plants. On the contrary, the concentration of H_2_O_2_ in transgenic lines was significantly lower than in the WT plants under non-stressed and cold stress conditions (Figure 5b). There were no changes in MDA contents between the WT and transgenic plants under normal conditions. However, after 12 h and 48 h of cold stress, the MDA concentration of WT plants was significantly higher compared to transgenic plants (Figure 5c). To further probe the role of the *OsIMP* gene in cold stress tolerance, transgenic lines and WT plants were analyzed for determination of antioxidant enzymes (superoxide dismutase (SOD), catalase (CAT) and peroxidase (POD)) activities for scavenging excessive reactive oxygen species (ROS). The results indicated that transgenic plants have significantly higher activities of the antioxidant enzymes SOD and CAT compared to that of wild type plants under non-stressed and cold stress conditions (Figure 6a,b). However, under normal conditions, we found that the POD activity was not significantly different between transgenic and WT plants (Figure 6c). Furthermore, after 12 h of cold stress treatment, sharp increases in the SOD, CAT and POD activities were observed with transgenic plants compared to wild type plants.

Furthermore, we compared the above biochemical parameters in the same plant lines under stress and non-stress conditions. The results revealed that chlorophyll content of WT plants significantly decreases after 24 h of cold stress treatment, whereas significant reduction from transgenic lines was observed over a period of 48 h (Figure S2a). In contrast, increased contents of MDA and H_2_O_2_ were found in WT plants after 12 and 24 h cold stress respectively, but transgenic lines showed significant increase only after 48 h cold treatment (Figure S2b,c), implying that the *OsIMP* transformed tobacco plants have improved cold tolerance. Additionally, antioxidant enzymes including SOD, CAT and POD, play key roles in protecting plants against oxidative stress by scavenging ROS; the activities of these enzymes were notably elevated in transgenic lines after cold treatment, and WT plants displayed a slight increase after cold stress (Figure S2d–f). 

## 3. Discussion

Cold stress frequently imposes an obviously negative influence on the growth and development of plants, including the decreased synthesis of total chlorophyll, as well as an increase in the amount of ROS, which leads to peroxidation of the cellular membrane lipids and overproduction of MDA. On the other hand, to withstand cold stress, the plants have evolved protective strategies to cope with the adverse environment by changing the expression of the stress response genes controlling the production of proteins and metabolites. For example, plants respond with improved antioxidant enzyme activities for scavenging ROS in addition to enhanced synthesis and accumulation of non-toxic compounds known as compatible solutes, such as soluble sugars, proline and inositol. In the present study, we obtained the full-length cDNA of the *OsIMP* gene coding IMPase in Gaogonggui. Expression profile analysis has found that cold stress and ABA treatment induce the expression of the *OsIMP* gene in rice, with these results suggesting that the *OsIMP* gene may play an important role in improving tolerance to cold stress. 

IMPase catalyzes the final step of inositol biosynthesis. Increasing evidence has also suggested that inositol and its derivatives were implicated in several physiological processes, including growth, development, and environmental stress in higher plants. In the present study, we isolated a full-length cDNA sequence of the IMPase coding gene in the cold-tolerant rice landrace in Gaogonggui from the Guizhou province of China, which is designated as *OsIMP*. The results of multiple alignment in conjunction with a search of CDD showed the enzyme active sites of IMPase required for metal and substrate binding, the IMPase characteristic domain ^88^DPLDGT^93^ (superscripts denote the residue number of the IMPase sequence) between strand β4 and helix α4, the ^66^GEET^69^ motif in helix α3 and the ^217^WDXAAG^222^ motif in helix α7 (Figure 1). These are indispensable for metal and substrate binding to IMPase. The mutagenesis analysis results have shown that Trp and Asp in the WDXAAG motif as well as Asp in the DPLDGT motif were key amino acid residues for lithium and magnesium affinity, with mutations in these residues almost completely abrogating IMPase activity [29]. In addition, Glu residues in GEET motif might be involved in activating water for nucleophilic attacks on the substrate [30]. Summarily, our multiple alignment results were consistent with the previous reports on IMPase from *Mycobacterium tuberculosis* and humans [31,32]. A phylogenetic tree constructed using the sequences of IMPase from Gaogonggui and other plant species have revealed that rice IMPase was most closely related to that of the wild rice *Oryza brachyantha* (Figure 2). 

Previous studies have also suggested that IMPase activity was strongly induced by abiotic stresses, including salt, cold, heat and drought in chickpea (*Cicer arietinum* L.) seedlings, indicating that IMPase may be implicated in the inositol-mediated abiotic stress adaptation in higher plants [26]. To explore whether *OsIMP* gene responds to cold stress in Gaogonggui, we examined the transcript level of this gene under cold stress conditions, with results having shown that *OsIMP* gene was significantly induced by cold treatment at 4 °C (Figure 3a). Furthermore, the phytohormone ABA was involved in the inducible transcription of cold-related genes and enhanced cold tolerance in plants [33,34]. To understand the effect of exogenous ABA treatment on the transcription of the *OsIMP* gene, qRT-PCR was carried out to detect the transcript level of the *OsIMP* gene. A significant increase in transcripts of this gene was observed in Gaogonggui seedlings treated by ABA (Figure 3b). These results revealed that the *OsIMP* gene may play a crucial role during the cold stress response by an ABA-dependent pathway in rice Gaogonggui. 

To gain full understanding of the molecular mechanism regulating the transcription of the *OsIMP* gene responding to cold stress and ABA treatment, we isolated a 2.2 kbp promoter sequence of the *OsIMP* gene from Gaogonggui. The analysis results of *cis*-acting elements have revealed that LTR and ABRE-elements, involved in low temperature and ABA responsiveness respectively, existed in the promoter sequence (Table 1). This might be the possible explanation for the response of the *OsIMP* gene to cold stress and ABA treatment. Furthermore, in cold-tolerant plant species, such as *Hordeum vulgare* and *Brassica napus*, LTR-elements were found in the promoter region of cold response genes and enhanced the expression of low temperature responsive genes under cold conditions [35,36].

To further probe the potential function of the *OsIMP* gene in cold stress tolerance, we successfully generated transgenic tobacco plants overexpressing the *OsIMP* gene by the *Agrobacterium*-mediated transformation method. Gas chromatography coupled with mass spectrometry (GC-MS) analysis has demonstrated that the synthesis of inositol in transgenic plant lines was significantly enhanced compared to WT tobacco plants. Low temperature is one of the most serious abiotic stress factors, inhibiting the synthesis of total chlorophyll and leading to overproduction of ROS [37]. The high concentration of ROS leads to oxidative damage to membrane lipids, resulting in enhanced production of MDA in cells [38]. Therefore, total chlorophyll, H_2_O_2_ and MDA are effective indicators of cold stress confronted by plants. In our work, cold stress inhibited the synthesis of total chlorophyll in both the transgenic and WT tobacco plants, but it was found that transgenic tobacco plants had a significantly higher content of total chlorophyll than WT plants, Sharma et al. [39] have reported that the total content of chlorophyll is closely correlated with cold tolerance in plants. Similarly, transgenic tobacco and rice plants displayed improved cold tolerance and had a significantly higher level of total chlorophyll compared to WT plants [40,41]. In contrast, higher contents of H_2_O_2_ and MDA were detected in WT plants in comparison to transgenic plants under room temperatures (26 °C) and cold stress conditions. These increased contents of H_2_O_2_ and MDA can impair metabolism by oxidative damage in plant cells [42]. On the basis of the above results, it can be noted that overexpression of the *OsIMP* gene conferred cold tolerance in transgenic plants, partially by decreasing production of H_2_O_2_ and MDA. This protects plants against oxidative damage under cold stress conditions. 

Because of their sessile lifestyle, plants need to develop wide adaptive strategies to survive against continuous exposure to all kinds of abiotic stresses. Among various strategies, the enzymatic antioxidant defense system plays an important role in protecting plant cells against oxidative damage by scavenging excessive ROS, which largely accumulated in plants under abiotic stress conditions. It has been demonstrated that cold tolerance is positively correlated with the increasing activities of antioxidant enzymes, such as SOD, CAT and POD in plants [43,44]. In the present work, the cold tolerance of transgenic tobacco plants overexpressing the *OsIMP* gene was further investigated by analyzing the enzyme activities of SOD, CAT and POD. Results have shown that *OsIMP* transgenic tobacco plants exhibited significantly enhanced activities of these antioxidant enzymes under normal and cold stress conditions. In particular, under cold stress conditions, sharp increases in antioxidant enzyme activities were observed in *OsIMP* transgenic tobacco plants, which possessed significantly higher enzyme activities than that of WT plants (Figure 6). It is suggested that overexpression of the *OsIMP* gene strengthens the activities of antioxidant enzymes and confers cold stress tolerance in tobacco plants. In other studies, it has also been reported that transgenic plants with higher activities of antioxidant enzymes had relatively improved cold stress tolerance in comparison with WT plants [45,46]. 

Inositol, as a cyclic polyol, is involved in enhancing stability of macromolecules and reducing the formation of hydroxyl radicals. Thereby, this protects the cellular membranes and biomacromolecule from oxidative damage under the abiotic stress conditions [47,48]. In addition, methylated derivatives of inositol, such as d-ononitol and d-pinitol, are implicated in improving salt stress tolerance in some halotolerant plant species [49,50]. Furthermore, inositol is an important precursor of the raffinose family oligosaccharides (RFOs), such as raffinose, stachyose, and verbascose, which accumulate in plants exposed to the abiotic stresses, such as cold, drought and high salinity [18,51,52]. Previously, Hincha [53] also found that RFOs play an important role in cellular membrane protection and in the antioxidant defense system. On the other hand, although transgenic tobacco plants overexpressing *OsIMP* gene in rice showed the improved cold tolerance, rice and tobacco are organisms that have different origins. In the future, additional work must be performed to reveal whether data on *OsIMP* obtained from transgenic tobacco plants can be also applied to rice. For example, this could include the generation of transgenic rice plants overexpressing *OsIMP* gene or decreasing the transcription level of the *OsIMP* gene in rice plants through the RNA interference (RNAi) technique. In summary, the above results indicated that overexpression of *OsIMP* gene lead to increased biosynthesis of inositol and improved antioxidant enzyme activities, thus conferring cold stress tolerance in transgenic tobacco plants. However, whether overexpressing *OsIMP* gene affects the biosynthesis of RFOs, d-ononitol and d-pinitol will be further researched in the future. 

## 4. Materials and Methods 

### 4.1. Plant Materials

Rice cultivated mature seeds from Gaogonggui were collected from the Congjiang county of Guizhou province in China. Seeds of the rice cultivation in Gaogonggui were surface-sterilized in 10% NaClO for 10 min, before being rinsed thoroughly with sterile water. Subsequently, they were soaked in water to accelerate germination at 37 °C for 48 h without illumination. They were transferred to plastic boxes filled with Hoagland nutrient solution and perlite substrate at a photo flux density of 350–450 μmol·m^−2^·s^−1^ at 70% relative humidity and at 28/25 °C with a 14 h photoperiod in a growth chamber. When the seedlings grew to the 4–6 leaf stage, the plastic boxes were divided into four groups. For the ABA treatment, the control group was sprayed with distilled water, while the treatment group was sprayed with 20 mg·L^−1^ ABA. For the cold treatment, the control group was maintained at room temperature (26 °C), while the treatment group was subjected to cold stress at 4 °C, with all other conditions remaining constant. Samples were collected from the full rice seedlings at different time points (0, 3, 6, 12, 24 and 48 h), instantly frozen in liquid nitrogen and stored in an ultra-low temperature refrigerator to be used for further analysis. At each time point, three biological replicates were conducted.

### 4.2. Methods

#### 4.2.1. Cloning and Bioinformatic Analysis of the *OsIMP* Gene

The expressed sequence tag of the *OsIMP* gene in Gaogonggui previously obtained from a subtractive cDNA library was compared with the GenBank database at the NCBI using the basic local alignment search tool to acquire a putative sequence of IMPase coding gene from the rice cultivation in Nipponbare (accession number: NM_001057109). A pair of gene specific primers, OsIMP-F and OsIMP-R (Table S1), flanking the coding region of the *IMP* gene were synthetized to isolate the cDNA sequence of the *OsIMP* in Gaogonggui. The total RNA was extracted from rice seedlings from Gaogonggui through the RNA-Solv^®^ Reagent method [54] using E.Z.N.A.^®^ Total RNA Kit I (OMEGA bio-tek, Norcross, GA, USA), before being reversely transcribed into cDNA using MultiScribe^TM^ Reverse Transcriptase Kit (Invitrogen, Shanghai, China) according to the manufacturer’s instructions. The 50 μL PCR mixture contained 10.4 μL sterile water, 25 μL 2 × GC buffer (including Mg^2+^), 2.0 μL each primer, 8 μL dNTPs mix (2.5 mM each), 0.6 μL LA Taq DNA polymerase (Takara, Dalian, China) and 2 μL template of cDNA. PCR amplification was performed in 35 cycles at 94 °C for 3 min, 94 °C for 40 s, 51 °C for 45 s and 72 °C for 4 min. The PCR products were detected by agarose gel electrophoresis, before being linked into a pGEM-T Easy vector (Promega, Madison, WI, USA) for DNA sequencing. 

The amino acid sequence of the IMPase protein was searched against the NCBI’s Conserved Domain Database to find the conserved enzyme active sites. The theoretical isoelectric point (pI) and molecular weight of the IMPase from Gaogonggui were predicted by the ExPASy tool. For the multiple alignment and phylogenetic tree construct, the sequences of IMPase from other plants were obtained from the NCBI database. The multi-alignment was accomplished by ClustalX2.0 program and ESPript3.0 tools, with the phylogenetic tree constructed by a neighbor-joining algorithm using MEGA5.2 software. 

#### 4.2.2. Expression Profile Analysis of the OsIMP Gene by Real-Time Quantitative PCR

The total RNA was extracted from Gaogonggui seedlings through the RNA-Solv^®^ Reagent method [55] using E.Z.N.A.^®^ Total RNA Kit I (OMEGA Bio-Tek, Norcross, GA, USA), before being reversely transcribed into cDNA with a MultiScribe^TM^ Reverse Transcriptase Kit (Invitrogen, Shanghai, China). PCR primer pairs (Table S1) were designed for rice *OsIMP* (OsIMPQ-F and OsIMPQ-R), control genes *actin* (Actin-F and Actin-R) and *Ubiquitin 5* (*UBQ5*) (UBQ5-F and UBQ5-R) using the online tool Primer Quest [54]. Quantitative Real-Time PCR was used to analyze the transcript level of the *OsIMP* gene in Gaogonggui treated by cold stress and exogenous. PCR amplification was performed in a 20 μL reaction mixtures, including 10 μL 2 × Power SYBR Green PCR Master Mix (Applied Biosystems, Foster City, CA, USA), 1 μL of 10 µM of each primer, 4 μL cDNA and ddH_2_O separately. Amplification reactions were conducted in a ABI 7500 PCR (Applied Biosystems, Foster City, CA, USA) with conditions at 94 °C for 4 min to denature samples, followed 30 cycles each of 30 s at 94 °C, 45 s at 56 °C, 1 min at 72 °C and a final extension of 72 °C for 5 min. The *actin* and *UBQ5* genes from rice were used as a reference, and the 2^−ΔΔ*C*T^ method [56] was used to analyze the transcript levels of the *OsIMP* gene. All PCR reactions were run in three biological replicates.

#### 4.2.3. Amplification of the OsIMP Gene Promoter and Cis-Acting Element Prediction

The CTAB method was used to extract the genomic DNA of the Gaogonggui seedlings. The promoter sequence of the Os*IMP* gene was retrieved from NCBI (Os03g0587000), based on the upstream sequence of start codon of the *OsIMP* gene. Sequence specific primers, OsIMPP-F and OsIMPP-R (Table S1), were designed to amplify a 2.2 kb promoter region of the *OsIMP* gene from Gaogonggui. The *cis*-acting element analysis was performed by employing the PlantCARE and PLACE database.

#### 4.2.4. Plasmid Construction, the Generation of OsIMP Transgenic Tobacco Plants and Molecular Comfirmation

The cDNA sequence of *OsIMP* from Gaogonggui was amplified using primer pairs harboring the KpnI and SpeI restriction sites (Table S1). The amplified product was cloned into a pTCK303 plant binary expression vector containing maize ubiquitin promoter and the terminator sequence of the nopaline synthase (*NOS*) gene. The resultant construct was named as pTCK303–OsIMP. Subsequently, the construct was verified by restriction analysis and DNA sequencing, before being inserted into the *Agrobacterium tumefaciens* strain LBA4404. The strain LBA4404 containing the construct pTCK303–OsIMP was transformed into tobacco (*Nicotiana tabacum* var. K326) using the *Agrobacterium*-mediated method [57]. The agar-solidified Murashige and Skoog medium containing 20 mg/L hygromycin was used to screen T_0_ generation transgenic plant lines. The putative transgenic tobacco plants were verified by PCR amplification with the primers HYG-F and HYG-R (Table S1), which were designed based on the DNA sequence of the hygromycin gene. This demonstrated the presence of the hygromycin gene in transgenic tobacco plants.

#### 4.2.5. Measurement of Inositol Content in WT and T1 Transgenic Tobacco Plants

Inositol content was measured by the GC-MS method [58,59] with minor modifications. A total of 20 mg of freeze-dried powder from tobacco leaves and 1 mL of solvent mixtures (MeOH, H_2_O and CHCl_3_) were transferred into a 5 mL centrifuge tube. This included 200 μL of 40 μg/mL ribitol as the internal reference, which was extracted for 40 min in the ultrasonic apparatus and centrifuged for 10 min at 15,000 *g*. A total of 500 μL of supernatant was transferred to a new 2 mL-tube, dried in a vacuum concentrator at room temperature. Derivatization of the dried extract was performed as in Roessner [60], while samples were analyzed by an Agilent 6890-5975 GC-MS system (Agilent, Santa Clara, CA, USA). An Automatic Mass Spectral Deconvolution and Identification System (AMDIS) (NIST, Gaitherburg, MD, USA) was employed to identify peak retention time and intensities of inositol and the internal reference, ribitol. The relative content of inositol was calculated according to the peak area ratio of the internal reference, ribitol, to inositol.

#### 4.2.6. Cold Stress Treatment and Determination of Stress-Associated Physiological Indicators

Seeds of tobacco plants (wild and transgenic type) were surface-sterilized with 70% ethanol and 2% (*v*/*v*) sodium hypochlorite solution, before being rinsed five times with deionized water. Subsequently, the seeds were sowed in plastic boxes containing Baltisches substrate nutrient soil (Hawita-Grupe GmbH, Vectha, Germany) for 30 days in a plant growth chamber maintained at 26 ± 2 °C under a 16 h light/8 h dark cycle with a photo flux density of 350–450 μmol·m^−2^·s^−1^ and 70% relative humidity. Before the cold stress treatment, PCR was performed to remove the segregating non-transgenic T_1_ plant lines. For cold treatment, the plastic boxes containing tobacco plants at the 4–6 leaf stage were transferred into a growth chamber at 2 °C for 48 h. 

Total chlorophyll concentration was analyzed through Qin’s method [61]. MDA content was assayed by Hodges’ method [62], which was briefly modified following the protocol of MDA assay kit (Suzhou Comin, Suzhou, China). For the measurement of H_2_O_2_, the method of Jana and Choudhuri [63] was adopted according to the instructions of the H_2_O_2_ assay kit (Suzhou Comin, Suzhou, China). All assays were performed using a DU640 spectrophotometer in three biological replicates.

Antioxidant enzyme activities assays were carried out as follows. A total of 0.1 g of the third or fourth leaf from the bottom to the top was harvested from each plant, which were immediately homogenized in a 50 mM K–phosphate extraction buffer containing 0.2 mM EDTA. The homogenate was centrifuged at 15,000 *g* for 10 min at 4 °C, before the supernatants were used as a crude enzyme extract for activity assays of antioxidant enzymes CAT, SOD and POD using commercial assay kits (Nanjing Jiancheng, Nanjing, China). The activities of the above enzymes were detected using the DU640 spectrophotometer (Beckman, Brea, CA, USA). Soluble protein content was analyzed by the Bradford method [64]. All measurements were performed in three biological replicates. 

## 5. Statistical Analysis

All data were derived from three biological replicates and were expressed as means ± SE. The significant difference between WT and transgenic plants were evaluated by Duncan’s tests using SPSS software (Chicago, IL, USA), which are indicated by asterisks (* *p* < 0.05, ** *p* < 0.01).

## Figures and Tables

**Table 1:** The motif prediction of the *OsIMP* gene promoter.

Code	Start	Strand	Motif	Function
ABRE	25	−	TACGGTC	abscisic acid responsiveness
Box-W1	1190	+	TTGACC	fungal elicitor responsive element
Box-W1	1838	−	TTGACC	fungal elicitor responsive element
CAAT-box	40	+	CAAAT	promoter and enhancer regions
CAT-box	1149	+	GGAGATG	part of a light responsive element
G-box	450	−	CACATGG	light responsiveness
GAG-motif	616	−	AGAGATG	part of a light responsive element
GARE-motif	273	+	AAACAGA	gibberellin-responsive responsive element
GARE-motif	1394	+	TCTGTTG	gibberellin-responsive responsive element
HSE	1085	−	AAAAAATTTC	heat stress responsiveness
LTR	2000	+	CCGAAA	low-temperature responsiveness
MBS	382	−	TAACTG	MYB Binding Site
TC-rich repeats	439	+	ATTTTATTCA	defense and stress responsiveness
TC-rich repeats	836	+	ATTTTCTTCA	defense and stress responsiveness
TC-rich repeats	1875	+	ATTTTCTTCA	defense and stress responsiveness
TC-rich repeats	1060	+	ATTTTCTTCA	defense and stress responsiveness
TC-rich repeats	2012	+	ATTTTCTTCA	defense and stress responsiveness
TCA-element	1041	−	CCATCTTTTT	salicylic acid responsiveness
TCA-element	2013	−	GAGAAGAAAA	salicylic acid responsiveness
TGA-element	35	+	AACGAC	auxin-responsive element

## Supplementary Materials

The following are available online at www.mdpi.com/2073-4425/8/7/179/s1. Figure S1: Analysis of *OsIMP*; Figure S2: Analysis of biochemical parameters in same plant lines under cold stress at different time points and Table S1: list of oligonucleotide primers used in this study.

## Abbreviations

IMPase	l-*myo*-inositol monophosphatase
LTR	low-temperature responsiveness
ABA	abscisic acid
MDA	malondialdehyde
SOD	superoxide dismutase
CAT	catalase
POD	peroxidase
MIPS	l-*myo*-inositol 1-phosphate synthase
RFOs	raffinose family of oligosaccharides
