# Supplementary material for: Overexpression of the OsIMP Gene Increases the Accumulation of Inositol and Confers Enhanced Cold Tolerance in Tobacco through Modulation of the Antioxidant Enzymes’ Activities
Source: Genes (Basel). 2017 Jul 20;8(7):179. doi: 10.3390/genes8070179 (PMC5541312; doi:10.3390/genes8070179)
Supplement: Supplementary file 1 [file genes-08-00179-s001.pdf]

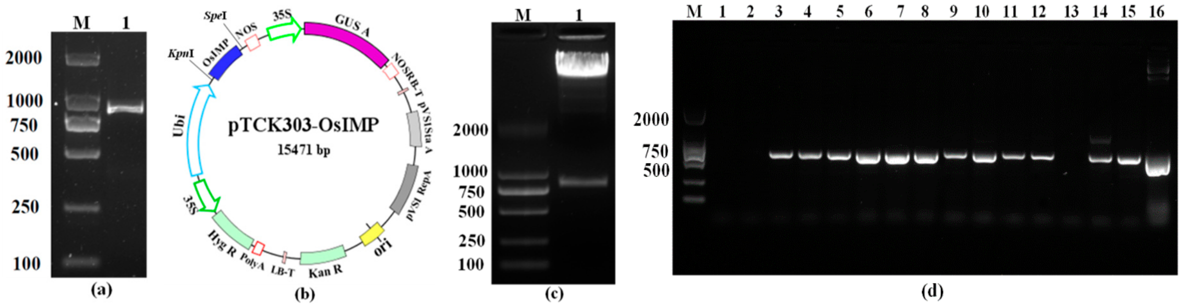

Figure S1. Analysis of *OsIMP*: (**a**) M: DL2000 marker (Takara), 1: PCR product of *OsIMP* gene; (**b**) Construct of pTCK303–OsIMP vector; (**c**) Restriction analysis of pTCK303–OsIMP vector, M: DL2000 marker (Takara), 1: pTCK303–OsIMP vector (Restricted with restriction enzymes *Kpn* I and *Spe* I); (**d**) PCR validation of transgenic tobacco plants, M: DL2000 marker (Takara), 1–15: putative T_0 transgenic tobacco plants, 16: positive control.

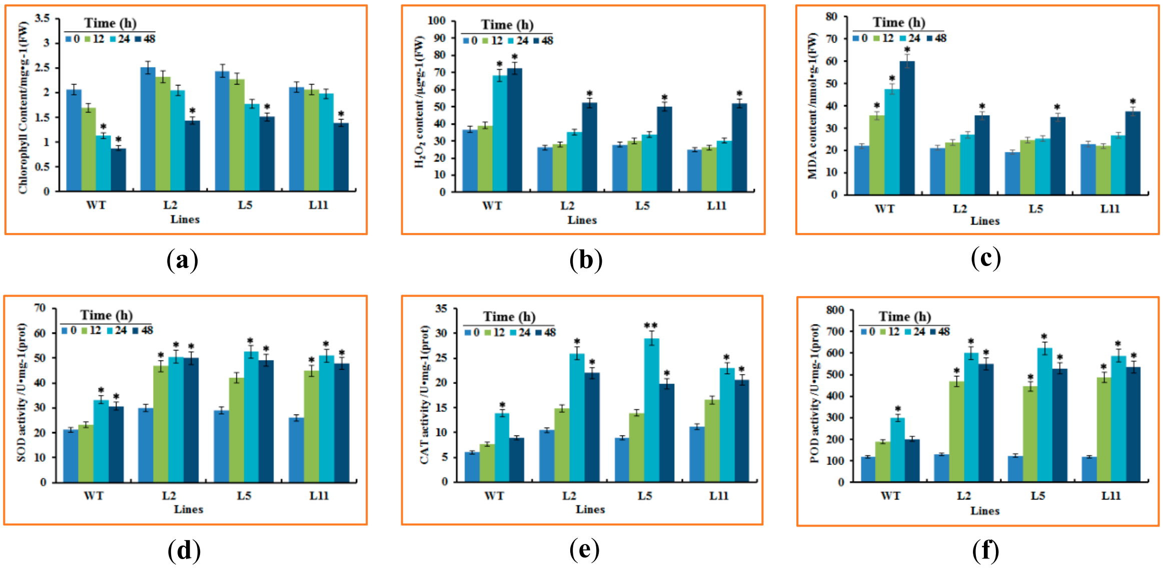

Figure S2. Analysis of biochemical parameters in same plant lines under cold stress at different time points. (**a**) chlorophyll, (**b**) H_2O_2, (**c**) MDA, (**d**) SOD, (**e**) CAT and (**f**) POD, WT: Wild type; L2, L5 and L11: transgenic lines. Data are the mean ± SE of three biological replicates, while asterisks indicate significant difference (* $p < 0.05$, ** $p < 0.01$) in comparison with 0 h time point.

Table S1. Oligonucleotide primers (restriction sites are shown underlined).

Primer Name	Genbank Accession	Sequence (5′–3′)
OsIMP-F	NM_001057109.1	cggGGTACCATGGCGGAGGAGCAGTTCC
OsIMP-R		ggACTAGTACTTGTATCTCCCAATTC
OsIMPQ-F		TGCCTTCCTCAATGGTTCTC
OsIMPQ-R		GTATCATCCAGAGTGGCCTTATC
Actin-F	XM_015774830	CAGCACATTCCAGCAGAT
Actin-R		GGCTTAGCATTCTTGGGT
UBQ5-F	AK061988	GCCTCACCTACGTCTACAAC
UBQ5-R		CGATTTCCTCCTCCTTCCTTAC
OsIMPP-F	Os03g0587000	gcGTCGAC AGTGCTGACTAGTGTACGAT
OsIMPP-R		cgGAATTCCGGAATTCACTCCCAATCTAT
HYG-F		AGATGTTGGCGACCTCGTATTGGG
HYG-R		GAGGGCGAAGAATCTCGTGCTTTC
